# Reactivity of the 2-Methylfuran Phase I Metabolite 3-Acetylacrolein Toward DNA

**DOI:** 10.1021/acs.jafc.4c07280

**Published:** 2024-11-04

**Authors:** Verena Schäfer, Simone Stegmüller, Hanna Becker, Elke Richling

**Affiliations:** Department of Chemistry, Division of Food Chemistry and Toxicology, University of Kaiserslautern-Landau, Kaiserslautern D-67663, Germany

**Keywords:** 2-methylfuran, 3-acetylacrolein, human liver, DNA adducts, primary rat hepatocytes

## Abstract

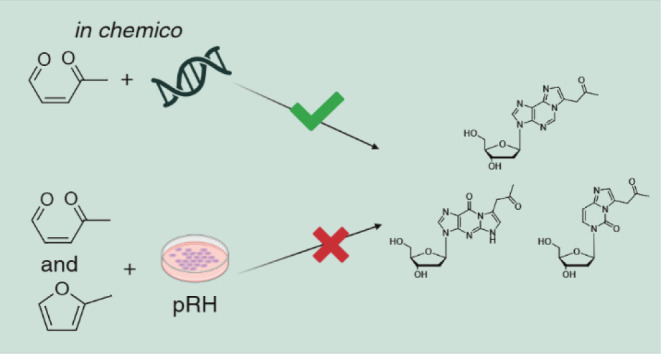

2-Methylfuran (2-MF) is a well-known industrial chemical and also formed via thermal treatment of food. One main source of 2-MF in the human diet is coffee. 2-MF is known to form 3-acetylacrolein (AcA, 4-oxopent-2-enal) via cytochrome P 450 metabolism and further reacts with amino acids in vivo. Still the reactivity toward other biomolecules is rather scarce. Therefore, AcA was synthesized, and its reaction with 2′-deoxyadenosine (dA), 2′deoxyguanosine (dG), 2′deoxycytosine (dC), and 2′-deoxythymidine (dT) was tested. For this purpose, adduct formation was performed by acid hydrolysis of 2,5-dihydro-2,5-dimethoxy-2-methylfuran (DHDMMF) as well as pure AcA. The structures of these adducts were confirmed by UPLC-ESI^+^-MS/MS fragmentation patterns and ^1^H-/^13^CNMR spectra. Except for dT, which showed no reactivity, all adducts of AcA were characterized, which enabled the development of sensitive quantification methods via (U)HPLC-ESI^±^-MS/MS. Pure AcA was synthesized by oxidation of 2-MF using dimethyldioxirane (DMDO), and its behavior in aqueous medium was studied. Incubations of AcA and isolated DNA of primary rat hepatocytes (pRH) showed time- and dose-dependent formation of the identified DNA adducts dA-AcA, dG-AcA, or dC-AcA. In contrast, the DNA adducts dA-AcA, dG-AcA, or dC-AcA were not detected on a cellular level when pRH were incubated with 2-MF or AcA. This indicates an efficient detoxification or reaction with biomolecules in the cell, although the induction of other DNA damage, possibly also by other metabolites, cannot be ruled out in principle.

## Introduction

2-Methylfuran (2-MF, CAS No. 534-22-5) has the molecular formula C_5_H_6_O with a molecular weight of 82.10 g/mol. At room temperature, 2-MF is a light yellow liquid with a chocolate-like odor, which has a boiling point of 63–64 °C. The chemical is highly flammable, has a density of 0.92 g/cm^3^ at 20 °C and a vapor pressure of 16 kPa. In industry, 2-MF is synthesized from furfural recently produced from lignocellulose to be used as biofuel via dehydroxygenation.^1^ Here the approach is the use of biomass to produce sustainable chemicals and fuels at an industrial scale. 2-MF is additionally used in industry as a starting material for further synthesis of, for example, chloroquine, methylfurfural and nitrogen- or sulfur-containing heterocycles. Research is also being conducted into the utilization of 2-MF as a fuel produced from biomass.^2−4^

For humans, exposure to 2-MF is most relevant as a food contaminant, whereby the thermal treatment of food or raw products plays a decisive role. The formation of high 2-MF levels has been attributed mainly to the so-called Maillard reaction. Model experiments showed that 2-MF can be formed from sugars such as d-glucose while retaining the carbon backbone via cyclization and dehydration. On the other hand, the contaminant can be formed during the thermal degradation of the amino acids l-alanine and l-threonine^5^ or from lipid peroxidation.^6,7^ 2-MF is found in foods such as coffee, cereals, and canned food in high amounts summarized by the EFSA CONTAM Panel^8^ and reported recently by others.^9−11^

Studies with rats show hepatotoxic effects of 2-MF in the form of necrosis and, to a lesser extent, damage to the lung tissue.^12^ Distribution and binding studies with radiolabeled 2-MF confirmed the liver as the main target organ.^13^ Meanwhile, the formation of a highly reactive α,ß-unsaturated phase I metabolite, namely 3-acetylacrolein (4-oxopent-2-enal, AcA, CAS-Nr. 5729-47-5; C_5_H_6_O_2_; *M* = 98.10 g/mol) is known. In the past, this metabolite was only indirectly trapped by disemicarbazon and postulated.^13^ More recently by our group the formation of AcA via CYP 2E1 was proofed using microsomes and primary rat hepatocytes (pRH) (see Schäfer et al. 2024).^14^ This reactive intermediate can react with nucleophiles such as proteins and DNA. In the classic Ames test, 2-MF with and without metabolic activation (using liver homogenate, S9 mix) showed no mutagenic potential apart from one ambivalent result. 2-MF was tested in *Salmonella* typhimurium strains at concentrations up to 1100 μmol/plate with negative results.^15^ Chromosome aberrations were detected in CHO cells after incubation with 2-MF with and without S9 mix (10–75 mM 2-MF without S9; 40–150 mM 2-MF with S9). However, the clastogenic potential proved to be lower with S9 mix or without the addition of NADPH to the S9 mix, which was interpreted as inhibitory effects of 2-MF on the enzyme system.^16^ No chromosomal aberrations were observed in vivo in bone marrow cells and spermatocytes of mice administered 100–400 mg/kg bw/d for 5 days. Huo et al. (2019, 2020)^17,18^ investigated the sub/acute genotoxicity of 2-MF in vivo in Sprague-Dawley rats in a 3-day and 28-day study using the comet assay, pig-a gene mutation test, and micronucleus test. For the former, the rats were treated with 25–150 mg/kg bw/d 2-MF p.o. for 3 days and peripheral blood was used for the tests. While a significant increase in tail intensity was observed in the alkaline comet assay in the highest dose group, no difference to negative controls was detected in the micronucleus test and pig-a gene mutation test.^17^ For the 28-day study, the rats were administered 0.625–20 mg/kg bw/d 2-MF p.o., with negative results in the alkaline comet assay (peripheral blood, liver), pig-a gene mutation test (peripheral blood), and micronucleus test (peripheral blood, bone marrow).^18^ The authors concluded that 2-MF is not expected to have an acute or subacute mutagenic effect in vivo.

In vivo distribution and binding studies with ^14^C-labeled 2-MF (50–200 mg/kg bw in Sprague-Dawley rats) showed the highest radioactivity bound to DNA isolated from the liver and to a lesser extent from the kidney.^19^ However, no specific adducts are known to date. Thus, neither a clear genotoxic or mutagenic effect of 2-MF could be proven nor a hypothesis regarding a mechanism could be established. Recently, a 90-day rat toxicity study has shown cholangiofibrosis and effects on the hepatobiliary system.^20^ The group has reported a NOAEL of 1.2 mg/kg/day for both sexes. The compound was administered intragastrically. From their results they stated that 2-MF is not involved in genotoxic events in the animal model used here.

In the frame of our investigations the in chemico reactivity of AcA toward DNA was investigated here. The respective compounds were synthesized to establish a stable isotope dilution analysis (SIDA) method for quantification. As a proof of concept, primary rat hepatocytes were incubated with 2-MF and AcA to determine potential DNA adducts.

## Materials and Methods

For use in synthesis, 2,5-dihydro-2,5-dimethoxy-2-methylfuran (97%, mixture of *cis* and *trans*, DHDMMF), acetic acid (99%) were from Avantor Performance Materials B.V. (Radnor, USA). For preparative purposes, HPLC grade formic acid and acetonitrile were purchased from Sigma-Aldrich (St. Louis, USA) and Fisher (Schwerte, Germany), respectively, whereas MS grade solvents formic acid and acetonitrile were purchased from Carl Roth (Karlsruhe, Germany) and Merck (Darmstadt, Germany), respectively. All other solvents and chemicals were of p.a. grade.

### Synthesis of 3-Acetylacrolein (AcA)

2-MF (BHT-stabilized for synthesis; 0.6752 mL; 7.275 mmol) was diluted in acetone (dried over K_2_CO_3_, 6.623 mL) to a 1 M solution and stirred with an equimolar amount of dimethyldioxirane (DMDO) (for synthesis see Adam et al. 1991;^21^ 97 mL; 7.275 mmol) for 45 min at room temperature (RT). Acetone was then distilled off at 70–80 °C. The residue was purified over flash silica gel using pentane:diethyl ether as solvent, the ratio of which was varied from 3:1 to 1:1 to 1:3. The separation of the product from byproducts was monitored by thin layer chromatography (TC, silica gel 60) (hexane:ethyl acetate, 1:3). The solvents were removed at 40 °C and completely evaporated overnight at RT. The yield was 596.0 mg (5.670 mmol, 78%, yellow, viscose liquid) Purity was 91% (^1^H-NMR).

AcA (98.10 g/mol, C_5_H_6_O_2_):

^1^H-NMR (400 MHz, CDCl_3_): δ [ppm] 10.09 (d, J = 7.1 Hz, 1H), 6.94 (d, J = 11.8 Hz, 1H), 6.10 (dd, J = 11.8, 7.1 Hz, 1H), 2.32 (s, 3H).

^1^H-NMR (400 MHz, DMSO-*d*_6_): δ [ppm] 9.94 (d, J = 6.9 Hz, 1H), 7.20 (d, J = 11.8 Hz, 1H), 6.22 (dd, J = 11.8, 6.9 Hz, 1H), 2.34 (s, 3H).

^13^C-NMR (151 MHz, DMSO): δ [ppm] 199.40, 193.08, 142.51, 136.53, 30.31.

HPLC-ESI^+^-MS/MS (0.5% HCOOH in H_2_O): MS^2^ [*m*/z] 116.96 [*trans-*AcA–OH_2_+H]^+^/ 89.0 [M–CO]^+^;

72.9 [M–COOH]^+^; 63.0 [C_2_O_2_H_6_]^+^.

^1^H-NMR signals of *cis*-AcA in different solvents is shown in Table S1.

### Synthesis and Characterization of DNA Adducts with AcA

#### 1-(3-((2R,4S,5R)-4-Hydroxy-5-(hydroxymethyl)tetrahydrofuran-2-yl)-3H-imidazo[2,1-I]purin-7- yl)propan-2-on (dA-AcA)

DHDMMF (354 μL; 500 μmol) was stirred with 3% acetic acid (833 μL) in 23.81 mL water for 1 h at 37 °C (500 rpm). 2′-Deoxyadenosine monohydrate (71 mg; 264 μmol) was dissolved in 25 mL of water and added to the acidic reaction mixture. The solution was stirred for 24 h at 37 °C and for a further 10 h after addition of 125 μL acetic acid (100%). The reaction mixture was by preparative HPLC, whereby the fractions analyzed by mass spectrometry were combined. Their volumes were reduced to ∼10 mL under reduced pressure (20 mbar; 1400 rpm; 30 °C) and the residue lyophilized (0.47 mbar; 15 °C). The yield was 27.003 mg (81.50 μmol, 31%; white solid) with a purity (^1^H-NMR) of 93%.

M (C_15_H_17_N_5_O_4_) = 331.33 g/mol:

^1^H-NMR (400 MHz, DMSO-*d*_6_): δ [ppm] 9.00 (s, 1H), 8.53 (s, 1H), 7.35 (s, 1H), 6.48 (t, *J* = 6.8 Hz, 1H), 5.37 (d, *J* = 4.1 Hz, 1H), 4.98 (t, *J* = 5.5 Hz, 1H), 4.48–4. 40 (m, 1H), 4.36 (s, 2H), 3.90 (q, *J* = 4.6 Hz, 1H), 3.66–3.49 (m, 2H), 2.75 (q, *J* = 13.2, 6.1 Hz, 1H), 2.37 (ddd, *J* = 13.2, 6.2, 3.3 Hz, 1H), 2.27 (s, 3H). Spectrum with structural assignment is shown in Figure S1 and Table S2.

^13^C-NMR (101 MHz, DMSO): δ [ppm] 204.45, 140.77, 139.91, 137.90, 135.88, 131.98, 123.11, 118.62, 88.03, 84.06, 70.78, 61.73, 37.97, 29.46. Structural assignment is shown in Table S2.

HPLC-ESI^+^-MS/MS: MS^2^ [*m*/*z*] 332.0/314.0 [M–H_2_O]^+^; 216.2 [M–dR]^+^; 186.0 [M–dR–CO_2_]^+^; 173.0 [M–dR–COCH_3_]^+^; 136.2 [dA+H]^+^. HPLC-ESI^+^-MS^2^ spectrum with corresponding *m*/*z* values is shown in Figure S2 and Table S3.

UV/Vis (H_2_O): λ_max_ = 225 nm (narrow); 275 nm (broad).

#### 3-((2R,4S,5R)-4-Hydroxy-5-(hydroxymethyl)tetrahydrofuran-2-yl)-7-(2-oxopropyl)-3*H*-imidazo[1,2-*a*]purin-9(5*H*)-one (dG-AcA)

DHDMMF (354 μL; 500 μmol) was stirred with 3% acetic acid (833 μL) in 23.81 mL water for 1 h at 40 °C (500 rpm). 2′-Deoxyguanosine hydrate [6 *m*-% H_2_O] (76 mg; 267 μmol) was suspended in 25 mL of water and completely dissolved in the acidic reaction mixture. The solution was stirred for 24 h at 37 °C and for a further 10 h after addition of 125 μL acetic acid (100%). The reaction mixture was by preparative HPLC, whereby the fractions analyzed by mass spectrometry were combined. Their volumes were reduced to ∼10 mL under reduced pressure (20 mbar; 1400 rpm; 30 °C) and the residue lyophilized (0.47 mbar; 15 °C). The yield was 25.885 mg (74.53 μmol, 28%; white solid) with a purity (^1^H-NMR) of 91%.

M (C_15_H_17_N_5_O_5_) = 347.33 g/mol:

^1^H-NMR (400 MHz, DMSO-*d*_6_): δ [ppm] 9.69 (s*), 8.07 (s, 1H), 7.15 (s, 1H), 6.27–6.17 (m, 1H), 5.30 (d, *J* = 4.0 Hz, 1H), 4.96 (t, *J* = 5. Five Hz, 1H), 4.42–4.33 (m, 1H), 4.13 (s, 2H), 3.83 (q, *J* = 4.5 Hz, 1H), 3.65–3.44 (m, 2H), 2.63–2.55 (m, 1H), 2.28–2.15 (m+s, 4H). Spectrum with structural assignment is shown in Figure S3 and Table S4.

HPLC-ESI^+^-MS/MS: MS^2^ [*m*/*z*] 348.0/330.2 [M–H_2_O]^+^; 232.2 [M-dR]^+^; 190.0 [M–dR–COCH_3_]^+^; 162.0 [M–dR–CH_2_COCH_3_]^+^; 152.0; 135.0 (fragments of ring moiety). HPLC-ESI^+^-MS^2^ spectrum with corresponding *m*/*z* values is shown in Figure S4 and Table S5.

UV/Vis (H_2_O): λ_max_ = 231 nm (narrow); 286 nm (wide).

#### 6-((2R,4S,5R)-4-Hydroxy-5-(hydroxymethyl)tetrahydrofuran-2-yl)-3-(2-oxopropyl)imidazo[1,2-*c*]pyrimidin-5(6*H*)-on (dC-AcA)

DHDMMF (354 μL; 500 μmol) was stirred with 3% acetic acid (833 μL) in 23.81 mL water for 1 h at 37 °C (500 rpm). 2′-Deoxycytosine monohydrate (81 mg; 330 μmol) was dissolved in 25 mL of water and added to the acidic reaction mixture. The solution was stirred for 24 h at 40 °C and for a further 10 h after addition of 125 μL acetic acid (100%). The reaction mixture was separated by preparative HPLC, whereby the fractions analyzed by mass spectrometry were combined. Their volumes were reduced to ∼10 mL under reduced pressure (20 mbar; 1400 rpm; 30 °C) and the residue lyophilized (0.47 mbar; 15 °C). The yield was 32.294 mg (105.09 μmol, 32%; white solid) with a purity (^1^H-NMR) of 99%.

M (C_14_H_17_N_3_O_5_) = 307.30 g/mol:

^1^H-NMR (400 MHz, DMSO-*d*_6_): δ [ppm] 7.63 (d, *J* = 8.0 Hz, 1H), 7.12 (s, 1H), 6.64 (d, *J* = 8.0 Hz, 1H), 6.32 (t, *J* = 6.8 Hz, 1H), 5.29 (d, *J* = 4.2 Hz, 1H), 5. 07 (t, *J* = 5.1 Hz, 1H), 4.31–4.24 (m, 1H), 4.15 (s, 2H), 3.82 (q, *J* = 3.5 Hz, 2H), 3.62–3.57 (m, 2H) 2.18 (s, 3H), 2.17–2.12 (m, 2H). Spectrum with structural assignment is shown in Figures S5, S6 and Table S6.

^13^C-NMR (101 MHz, DMSO): δ [ppm] 204.46 (CO), 146.88, 145.22, 132.68, 127.92 (aromatic carbon), 123.09 (aromatic carbon), 98.94, 87.71, 84.80, 70.45, 61.31, 40.14 (CH_2_), 39.83, 29.37 (CH_3_). Structural assignment is shown in Table S6 and Figure S6.

HPLC-ESI^+^-MS/MS: MS^2^ [*m*/*z*] 308.2/192.0 [M–dR]^+^; 179.8; 150.0 [M–dR–COCH_3_]^+^; 135.8 [M–dR–CH_2_COCH_3_]^+^; 121.0. HPLC-ESI^+^-MS^2^ spectrum is shown in Figure S7.

UV/Vis (H_2_O): λ_max_ = 276 nm (narrow), 217 nm (broad).

#### [^15^*N*_5_]-1-(3-((2R,4S,5R)-4-Hydroxy-5-(hydroxymethyl)tetrahydrofuran-2-yl)-3H-imidazo[2,1-i]purin-7-yl)propan-2-on (^15^*N*_5_-dA-AcA)

[^15^*N*_5_]-2′-Deoxyadenosine monohydrate (0.828 mg; 2.997 μmol) was dissolved in 600 μL water and DHDMMF (5.19 μL; 35.87 μmol) and 3% aqueous acetic acid (12.22 μL) were added. The solution was stirred for 24 h at 37 °C and for a further 10 h after addition of 25% aqueous acetic acid (5.9 μL). The reaction mixture was separated by preparative HPLC, whereby the fractions analyzed by mass spectrometry were combined. Their volumes were reduced to ∼10 mL under reduced pressure (20 mbar; 1400 rpm; 30 °C). The yield was 3.2 μg (9 pmol; 0.33%), determined via calibration (UV/Vis, MS) of the nonisotope-labeled adduct, as the amount of isotope-labeled reactant used was too small for gravimetric determination of the product.

M (C_15_H_17_^15^*N*_5_O_4_) = 336.29 g/mol:

HPLC-ESI^+^-MS/MS: MS^2^ [*m*/*z*] 337.2/319.2 [M–H_2_O]^+^; 221.0 [M–dR]^+^; 191.0 [M–dR–CO_2_]^+^; 179.2 [M–dR–COCH_3_]^+^; 141.0 [dA+H]^+^. Corresponding *m*/*z* values are shown in Table S3.

UV/Vis (H_2_O): λ_max_ = 225 nm (narrow); 275 nm (broad).

#### [^15^*N*_5_]-3-((2R,4S,5R)-4-Hydroxy-5-(hydroxymethyl)tetrahydrofuran-2-yl)-7-(2-oxopropyl)-3H-imidazo[1,2-*a*]purin-9(4H)-on (^15^*N*_5_-dG-AcA)

[^15^*N*_5_]-2′-deoxyguanosine monohydrate (0.726 mg; 2.502 μmol) was dissolved in 600 μL water and DHDMMF (5.19 μL; 35.87 μmol) and 3% aqueous acetic acid (12.22 μL) were added. The solution was stirred for 24 h at 37 °C and a further 10 h after addition of 25% aqueous acetic acid (1.2 μL). The reaction mixture was separated by preparative HPLC, whereby the fractions analyzed by mass spectrometry were combined. Their volumes were reduced to ∼10 mL under reduced pressure (20 mbar; 1400 rpm; 30 °C). The yield was 20.8 μg (59 pmol; 2%), determined via calibration (UV/Vis) of the nonisotope-labeled adduct, as the amount of isotope-labeled reactant used was too small for gravimetric determination of the product.

M (C_15_H_17_^15^*N*_5_O_5_) = 352.29 g/mol:

HPLC-ESI^+^-MS/MS: MS^2^ [*m*/*z*] 353.2/335.0 [M–H_2_O]^+^; 237.0 [M–dR]^+^; 195.0 [M–dR–COCH_3_]^+^; 167.0 [M–dR–CH_2_COCH_3_]^+^; 139.0 (fragment of ring moiety). Corresponding *m*/*z* values are shown in Table S5.

UV/Vis (H_2_O): λ_max_ = 231 nm (narrow); 286 nm (wide).

### Purification of DNA Adducts and Stable Isotopically Labeled Analogues

The reaction mixture for the synthesis of the DNA adducts was semipreparatively processed by HPLC-DAD using a fraction collector and the fractions were analyzed by mass spectrometry in the Q1 scan (UPLC-MS/MS see below). HPLC was performed with an Agilent 1200 Series with diode array-detector (DAD; Agilent Technologies, Santa Clara, California, USA) and conditions were as follows: VDSphere PUR C18-SE 5 μm column (250 mm × 20 mm, VDS Optilab, Berlin, Germany); solvent system: A water, B acetonitrile; gradient profile: from 0 to 2 min 5% B, then within 13 min to 20% B and within 0.5 min to 50% B and afterward isocratic 50% B for 2.5 min, then from 50% B within 0.5 min to 5% B and isocratic at 50% B for 3.5 min; flow rate 10 mL/min; injection volume 10 mL, membrane filtered (0.45 μm) prior injection; detection wavelength 254, 270, and 300 nm (reference wavelength 360 nm); as the retention times were 9 min for all compounds, every min a fraction was collected from 5 to 15 min.

### Reactivity of AcA with Nucleosides

In preliminary tests, potential reaction products of AcA with dA, dG, dC, and dT were analyzed using acid hydrolysis of DHDMMF. If necessary, the adducts were synthesized, characterized and quantification methods were established. After synthesis of AcA by DMDO oxidation, the reactivity of isolated AcA with the reactants was tested again in different buffer systems with varying pH values, focusing primarily on the conditions of in vitro studies (Table S7).

### Reactivity of AcA with Isolated DNA

The reactivity of AcA (obtained from the DMDO oxidation of 2-MF) with isolated DNA was investigated. For this purpose, isolated DNA from untreated pRH was incubated with 0.1–1000 μM AcA in a time- and dose-dependent manner. PRH were achieved by the procedure described in Schäfer et al. 2024.^14^ After 3 h of growth, cells were washed with PBS and stored directly at −80 °C until DNA isolation (see below).

While the stock solutions of AcA (0.01 to 1000 mM) were prepared in DMSO due to the solubility and stability of AcA, an intermediate dilution in water had to be carried out in order to achieve a constant DMSO concentration of 0.1% DMSO in the reaction solution. The final concentration could not be exceeded, as otherwise the necessary precipitation of the DNA to remove AcA would be prevented.

The incubation (0.01–1000 μM) was carried out in water or with K_2_HPO_4_ buffer (0.05 M in reaction solution). The final concentration of the buffer was achieved by adding 10 μL stock solution (0.5 M; pH 7.4) per 100 μL reaction solution. The addition of water was reduced accordingly.

In addition, 2, 10, or 20 μg salmon DNA dissolved in 100 μL water without addition of AcA was incubated and processed as follows.

The reaction mixture (Table S8) was incubated for 1, 6, 18, 24, or 48 h (37 °C, 650 rpm) in a 2 mL reaction vessel and stopped by precipitation with 1.8 mL ethanol (100%). The DNA was purified (from precipitation of the DNA), enzymatically hydrolyzed and measured.

### Reactivity of 2-MF or AcA with Primary Rat Hepatocytes (pRH)

All used pRH were achieved by the procedure described in Schäfer et al. 2024.^14^ The pRH were incubated with 2-MF (10–1000 μM) or AcA (0.1–5 μM) (*n* = 3) and stored at −80 °C until further processing.

### DNA Isolation

The pRH stored at −80 °C in cell culture dishes (100 mm) were thawed on ice, dissolved with 800 μL lysis buffer using a cell scraper and transferred to a 2 mL reaction vessel containing 15 μL protein kinase K solution. The sample was carefully mixed with 5 μL RNase solution and lysed for 4 h at 55 °C in a shaking incubator. Afterward, DNA was isolated as follows.^22^ The lysate cooled to room temperature was vortexed with 0.9 mL extraction solution 1 for 10 s. The sample was then centrifuged (14,000 rpm, 15 min, 4 °C, with brake) and the aqueous supernatant was transferred to a new 2 mL reaction tube. Incubation with 5 μL RNase solution was carried out for 20 min at room temperature. The second extraction with 0.8 mL extraction solution 2 was also vortexed for 10 s and centrifuged (14 000 rpm, 15 min, 4 °C, with brake) to transfer the aqueous supernatant again into a fresh 2 mL reaction tube. To remove organic residues, the sample was gently swirled with 1.2 mL ethanol (100%, −20 °C), causing the DNA to visibly sediment as fine, white threads.

After centrifugation (15,000 rpm, 15 min, 4 °C) and complete discarding of the supernatant, the DNA pellet was redissolved in 250 μL autoclaved H_2_O. The second precipitation was achieved by adding 50 μL sodium acetate solution (3 M) and 500 μL isopropanol (100%, −20 °C). The sample was centrifuged again (15,000 rpm, 15 min, 4°C) and the supernatant discarded to wash the DNA with ethanol (70%, −20 °C). To obtain the purified DNA, it was centrifuged (15,000 rpm, 15 min, 4 °C), the solvent was removed, the residue was dried at room temperature and then homogeneously dissolved in 50 μL autoclaved H_2_O.

Unless otherwise noted for enzymatic incubations or lyses, it proved advantageous to work on ice. In this way, a sharp phase separation could be maintained during extraction, even with a large number of samples with longer standing times, and clear precipitation of the DNA could be achieved. In the case of incomplete precipitation, precipitation of the DNA was made possible by storing the sample at −20 °C overnight, increasing the proportion of organic solvent and adding additional sodium acetate solution (3 M).

The DNA content was determined photometrically (Nanodrop) at λ_absorbance_ = 260 nm as ng/μL. Two absorption ratios made it possible to test the purity of the sample with regard to organic residues as well as RNA. The ’260/280’ value, i.e., λ_Absorbance_(260 nm)/λ_Absorbance_(280 nm), should be 1.8–1.85, while the absorbance ratio of 260 nm to 230 nm (’260/230’) between 1.8–2.2 was considered pure DNA.

### DNA Hydrolysis

Depending on the amount of expected DNA adducts, 30 μg or 50 μg of DNA in solution was adjusted to 135 μL with H_2_O in a 1.5 mL reaction vessel. For the determination by stable isotope dilution analysis, ^15^*N*_5_-labeled dA-AcA and dG-AcA of 0.75 nM and 5 nM were added to the sample as an internal standard. In addition, ^15^*N*_5_-labeled dG with a final concentration of 20 μM was used as an internal standard to determine the hydrolysis rate via the dG content.

Initially, according to Schumacher et al. (2013),^23^ the sample was incubated with 39 μL succinate buffer and micrococcal nuclease (53 mU per 1 μg DNA) for 24 h at 37 °C at 100 rpm. Subsequently, 93 μL Tris-buffer was thoroughly resuspended in the sample so that phosphodiesterase II (2.1 mU per 1 μg DNA) and alkaline phosphatase (0.6 mU per 1 μg DNA) further degraded the DNA overnight (37 °C, 100 rpm). On the third day, the proteins were precipitated using 500 μL ethanol (100%, −20 °C) and the sample was centrifuged (20 800 *g*, 15 min, 4 °C). The supernatant was concentrated in a new 1.5 mL reaction vessel using a vacuum centrifuge (20 mbar, 1400 rpm, room temperature) to remove the ethanol and water until a highly viscous residue was obtained.

### Measurements of DNA Adducts by UPLC-ESI-MS/MS Analysis

After AcA was incubated individually with the four nucleosides, the reaction products of AcA with isolated DNA were to be quantified. For this purpose, the isolated DNA was enzymatically hydrolyzed after incubation. For the quantification of in vitro samples, pRH were processed as described in Schäfer et al. 2024^14^ before they could be quantified using the following method. Chemicals of HPLC-MS grade purity were used for preparations. For separation and quantification, a UPLC-MS/MS system of AB Sciex was used. The quantification was performed with an Agilent 1290 Infinity system equipped with a degasser (G1379B), binary pump (G4220A), autosampler (G4226A) and column oven (G1330B) (Agilent Technologies, Santa Clara, California, USA) coupled to a QTRAP 5500 mass spectrometer (Sciex, Darmstadt, Germany). The latter was equipped with an electrospray ionization source (ESI), operating in the multiple reaction mode (MRM) with positive electrospray ionization (ESI^+^). The ESI-MS conditions were as follows: ion spray voltage (IS) 4500 V; curtain gas (CUR) 25 psi; nebulizer gas 55 psi; heater gas 60 psi; temperature (T) 450 °C. HPLC conditions were as follows: Acquity UPLC BEH Amide column; 1.7 μm (2.1 × 50 mm Waters Milford, USA) and a guard column (1.7 μm); solvent system: A 0.1% formic acid in water, B acetonitrile with 0.1% formic acid; gradient profile: isocratic 10% B for 1.5 min at 400 μL/min, then to 200 μL/min and, from 10 to 80% B over 0.5 min isocratic with 80% B for 3.0 min, from 80 to 20% B over 0.5 min and isocratic 20% B for 6 min at a flow rate of 400 μL/min; injection volume 2.0 μL; column oven temperature 30 °C. Membrane filtered (0.45 μm) prior injection; retention times were 0.7 min for dC-AcA, 0.9 min for dA-AcA and ^15^*N*_5_-dA-AcA, and 1.0 min for dG-AcA and^15^*N*_5_-dG-AcA, respectively. ESI^+^-MS/MS in the MRM method in positive mode was used to detect the respective compounds (Table S9).

To determine the content of the three DNA adducts dA-AcA, dG-AcA, and dC-AcA, calibration series were prepared in the concentration range of 0.01–20 nM. The internal standards used were^15^*N*_5_-dA-AcA (ISA) and^15^*N*_5_-dG-AcA (ISG) with final concentrations of 0.6 nM and 4.7 nM, respectively. The analytes were measured together with the internal standards solved in the initial solvent composition (90% MeCN; 10% water with 0.1% formic acid). All used dilutions were prepared in water.

Based on the calibration series, the regression lines with associated validation parameters were obtained. The validation parameters described a valid and robust method for the detection of dA-AcA and dG-AcA in the relevant concentration range of 0.05–20 nM. The distribution of the residuals was unremarkable. dC-AcA was determined semiquantitatively due to interference in all three mass transitions.

### Method Validation

The precision of the method for dA-AcA (0.05–20 nM), dG-AcA (0.05–20 nM) and dC-AcA (1–20 nM) were determined by intra- (five replicate analysis of one concentration in a row) and interday (one concentration on 5 days in a row) repetition experiments; the intraday coefficient of variation were for dA-AcA 2.3%, for dG-AcA 2.7%, and for dC-AcA 2.2%. For the interday experiments for dA-AcA 4.8%, for dG-AcA 4.4%, and for dC-AcA 3.4%. With the limit of detection (LOD) and the limit of quantification (LOQ), the analyte concentration was specified via the peak height with the 3-fold, 6-fold or 10-fold signal-to-noise ratio (S/N). While the LOQ was usually used as the smallest concentration of the calibration curve, the LOD was mainly determined by calculation. They were determined for dA-AcA (LOQ 0.05 nM, LOD 0.01 nM), dG-AcA (LOQ 0.05 nM, LOD 0.01 nM) and dC-AcA (LOQ 0.07 nM, LOD 0.02 nM; noise corresponds to peak height at 0.001 nM). The calibration approach adapted from the European Commission/Joint Research Centre was used.^24^

The content of the DNA adducts as a substance concentration was done by using regression lines based on the ratio of the area under curve (AUC) of the analyte to the internal standard. The amount of DNA or hydrolysis rate used was used to indicate the content in number of adducts per 10^8^ nucleosides. To calculate the hydrolysis rate, the dG content was determined assuming a guanosine-cytosine content of 44% in pRH DNA and 41% in salmon DNA. The method according to Stegmüller et al. (2018)^25^ was used to determine the hydrolysis rate of isolated DNA via the quantification of dG.

Shortly before measuring the sample, the viscous residue was resuspended in 50 μL of the initial superplasticizer composition (90% MeCN; 0.1% formic acid; in H_2_O). The suspension was centrifuged (13 000 *g*, 20 min, 4 °C) and the supernatant was transferred to a 1.5 mL vial with 200 μL insert. The samples were measured using the established UHPLC-ESI^+^-MS/MS method to determine the three DNA adducts dA-AcA, dG-AcA and dC-AcA using SIDA.

The conversion of the substance quantity-related content to the adduct content per 10^8^ nucleosides was made possible using the dG determination method.

To determine the recovery rate, dA-AcA, dG-AcA and dC-AcA were added to the sample matrix in concentrations of 1, 8, 10, and 12 nM (limit value, 80%, 100%, 120% average expected values). The spiked samples were added after DNA hydrolysis. The recovery rate was 100 ± 9% for dA-AcA and 92 ± 4% for dG-AcA. The semiquantitative recovery of dC-AcA was 114% but is only indicative.

## Results

The in silico studies using ToxTree and QSAR software suggested a potentially toxic character of 2-MF after metabolic activation (data not shown). The proposed metabolites were divided into two groups, one group derived from AcA and one from furfurylalcohol (FFA). AcA can be formed from 2-MF by oxidative ring opening.^13,14^ Similarly, the metabolic activation of furan to *cis*-2-butenedial (BDA) as a highly reactive metabolite has already been demonstrated and reported in literature.^26−28^ The same metabolic activation has been described for 2,5-dimethylfuran (DMF).^29^ Yet no evidence for the hydroxylation of the methyl group to FFA in phase I metabolism is yet known. Therefore, the focus was placed on AcA as the primary intermediate.

### Synthesis and Characterization of AcA and its DNA Adducts

AcA was synthesized via two routes, which differed in their synthesis steps and purity. The faster and more easily accessible route was via the release of AcA from the acidic hydrolysis of 2,5-dihydro-2,5-dimethoxy-2-methylfuran (DHDMMF) as reported before (see Schäfer et al. 2024).^14^ The equilibrium on the reactant side did not allow the isolation of AcA, but the addition of a reactant shifted the equilibrium on the product side. However, in order to investigate the reactivity in vitro, AcA was prepared as a pure substance. For this purpose, AcA was synthesized by oxidation of 2-MF using dimethyldioxirane (DMDO), which was more time-consuming and associated with a lower yield but higher purity. The product was characterized and tested for its stability and behavior in various solvents. A purity of 91% was determined in organic solvent (via ^1^H-NMR spetrum, DMSO-*d*_6_, stored at −20 °C). The oxidation of 2-MF via DMDO led to ring opening of the aromatic compound, with the cis-isomer of AcA as the selective product of the synthesis. The literature comparison of the ^1^H-NMR of the AcA synthesis by Ravindranath et al. (1984)^13^ showed a good correlation of the signals and coupling constants.

The stability of AcA was tested in aqueous solution. In the ^1^H-NMR spectrum (600 MHz, D_2_O), three main products were detected in a ratio of 1:1:0.2.

Table S1 provides an overview of the compounds described with structurally assigned ^1^H-NMR signals. The different configuration of the methine proton to the methyl group (or the position of the two hydroxyl groups relative to each other) was labeled isomer I and II.

### Reactivity of AcA with Nucleosides

As monomers of the DNA the four nucleosides dA, dG, dC, and dT were used as reactants. Initially, AcA from the acidic hydrolysis of DHDMMF was reacted separately with the four reactants and the synthesis of the respective reference substances were established based on the adducts formed. Except for dT, adduct formation was observed with dA, dG, and dC. The incubation of 2-MF with the reactants showed that such a reaction could be ruled out, as only trace amounts of reaction products were formed.

**Figure 1:**
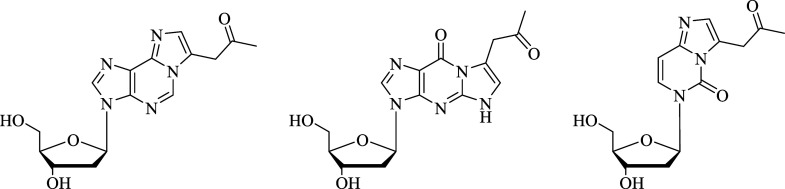
Structures of dA-AcA (left), dG-AcA (mid), and dC-AcA (right). AcA: 3-acetylacrolein, dA: 2′-deoxyadenosine, dC: 2′-deoxycytosine, dG: 2′-deoxyguanosine.

**Figure 2:**
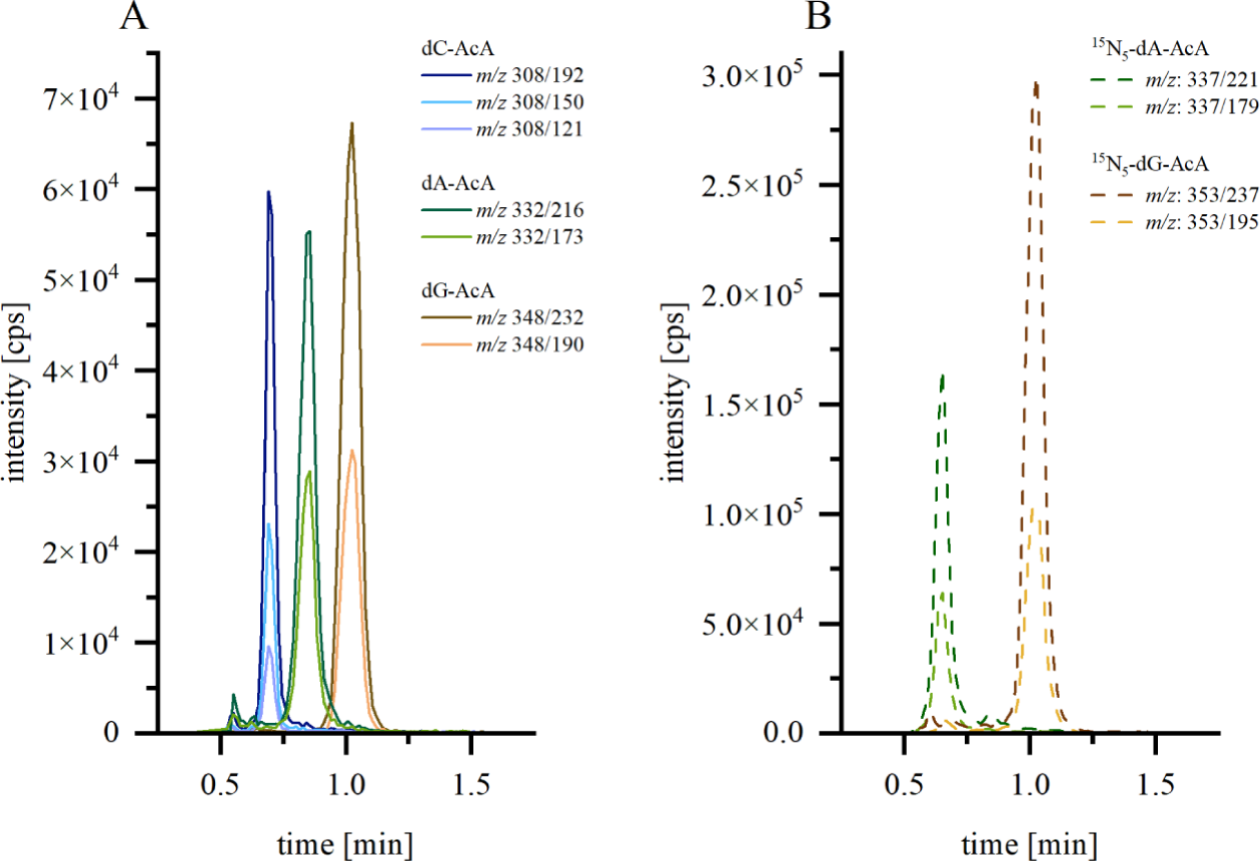
HPLC-ESI^+^MS/MS chromatogram (MRM mode) of dA-AcA (green), dG-AcA (yellow) and dC-AcA (blue) as well as the internal standards ^15^N_5_-dA-AcA (dashed, green) and ^15^N_5_-dG-AcA (dashed, yellow). Each 10 nM substance was measured using the method described in the material and methods section. AcA: 3-acetylacrolein, dA: 2′-deoxyadenosine, dC: 2′-deoxycytosine, dG: 2′-deoxyguanosine, *m*/*z*: mass-to-charge ratio.

Consequently, the synthesis of AcA and the respective adducts with, dA, dG, and dC, ^15^*N*_5_-dA-AcA, and ^15^*N*_5_-dG-AcA were performed to use internal standards for quantification (structures see Figure 1). The purity of the synthesis was primarily determined via ^1^H-NMR measurements and measured for further characterization via ^13^C-NMR spectroscopy. All synthesis products were characterized using liquid chromatography coupled with tandem mass spectrometry (HPLC-MS/MS). The first step was to confirm the molar mass via full scan and the chromatogram combined with the associated spectrum to further assess the purity. The production scan (MS^2^) with additional different fragmentation conditions was used to identify and confirm structural features. It formed the basis for the development of sensitive quantification methods in multiple reaction monitoring (MRM) mode. Figure 2 shows the HPLC-ESI^+^-MS/MS chromatogram (MRM mode) of dA-AcA, dG-AcA and dC-AcA, as well as the internal standards ^15^*N*_5_-dA-AcA and ^15^*N*_5_-dG-AcA (dashed lines), each with 10 nM substance.

To validate the quantification methods, the intra- (run-to-run) and interday (day-to-day) variability of the analytes was determined. All methodological parameters and their validation are listed in material and method section.

A sensitive UHPLC-MS/MS method was developed for the joint determination of the three DNA adducts (dA-AcA, dG-AcA, and dC-AcA). For the synthesis, DHDMMF was acid hydrolyzed with the addition of the respective nucleoside. Afterward the products were characterized by HPLC-MS/MS, UV/Vis, ^1^H and ^13^C-NMR (see Supporting Information). The corresponding stable isotopically labeled standards, ^15^*N*_5_-dA-AcA and ^15^*N*_5_-dG-AcA were successfully synthesized as well. A sensitive UHPLC-ESI^+^-MS/MS method was developed for simultaneous quantification of the three nucleoside adducts.

### Reactivity of AcA with Isolated DNA

In the next step, the reactivity of AcA toward isolated DNA was analytically characterized in a time- and dose-dependent manner. The aim here was to analyze the reactivity of AcA toward double-stranded (isolated) DNA as well as the qualitative and quantitative occurrence of the three adducts. The content was also specified as adducts per 10^8^ nucleosides. For this purpose, it was necessary to determine the hydrolysis rate of the DNA via the content of dG. Initially, commercially purchased salmon DNA (available in isolation) was used to test the reactivity of AcA. However, unexpectedly high levels of all three adducts were found in the untreated negative control (nontreated salmon DNA). The dependence of the detected DNA adducts on the amount of untreated salmon DNA used is shown in Figure S8 and correlated with an R^2^ between 0.999 and 1. If the levels were normalized to 10^8^ nucleotides, there were, as expected, no differences between the amounts of DNA used (2, 10, 20 μg). Considering the average occurrence of the base pairs GC (44%) and AT (66%) in salmon DNA, an adduct content of 4 *n*-% dA-AcA and 0.3 *n*-% dG-AcA was determined for the total nucleoside content. The semiquantitative determination of 19 *n*-% dC-AcA indicates a high proportion of AcA binding to dC.

As the negative control of untreated salmon DNA already contained adducts, DNA from primary rat hepatocytes (pRH) was used next. To investigate the reactivity of AcA toward DNA, the DNA of untreated pRH were isolated and purified. No corresponding DNA adducts, dA-AcA, dG-AcA, or dC-AcA, were detectable in the untreated control.

**Figure 3:**
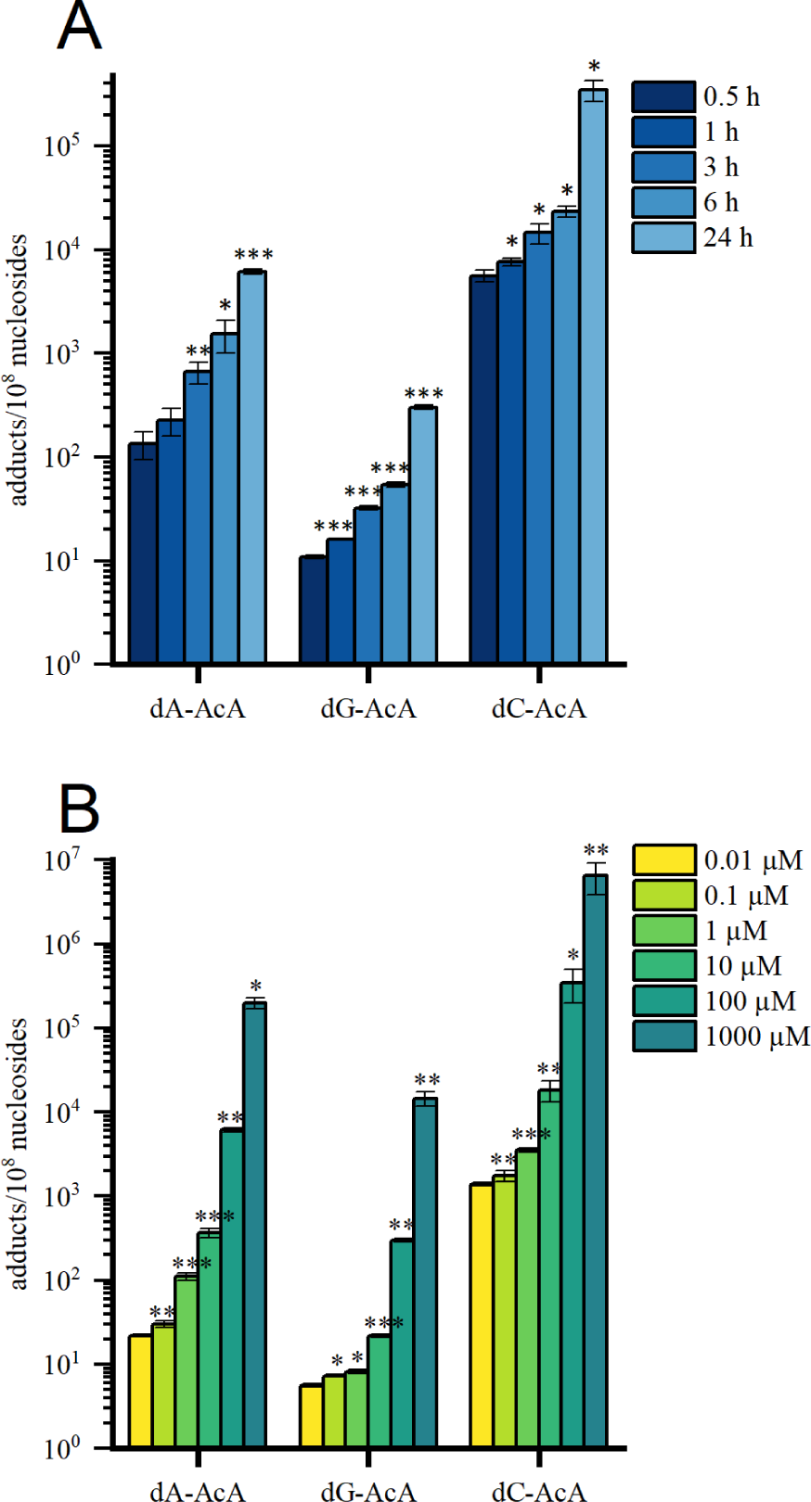
(A) Time- and (B) dose-dependent formation of dA-, dG- and dC-AcA after incubation of isolated DNA (20 μg) from pRH with AcA. The incubations at 37 °C were performed with a constant 100 μM AcA in the case of the time-dependent test (A), while the dose-dependent tests were incubated for a constant 24 h (B). *n* = 3, mean ± standard deviation. Significances were tested against the next lower unit using unpaired one-tailed *t* test with (*) *p* < 0.05 significant, (**) *p* < 0.01 highly significant, (***) *p* < 0.001 highly significant. AcA: 3-acetylacrolein, dA: 2′-deoxyadenosine, dC: 2′-deoxycytosine, dG: 2′-deoxyguanosine.

When isolated DNA of pRH was incubated with AcA (24 h with 0.01–1000 μM AcA or 0.5–24 h with 100 μM AcA), all three adducts were detected and their formation was significantly and linearly dose- and time-dependent (see Figure 3). After 24 h of incubation with a concentration of 100 μM AcA, the content of 6.14 × 10^3^ dA-AcA/10^8^ nucleosides exceeded that of dG-AcA (3.02 × 10^2^/10^8^ nucleosides) by a factor of 20. The highest adduct levels were determined for dC-AcA, but the 32-fold higher adduct levels for dA-AcA must be evaluated with reservations due to the semiquantitative method.

**Figure 4:**
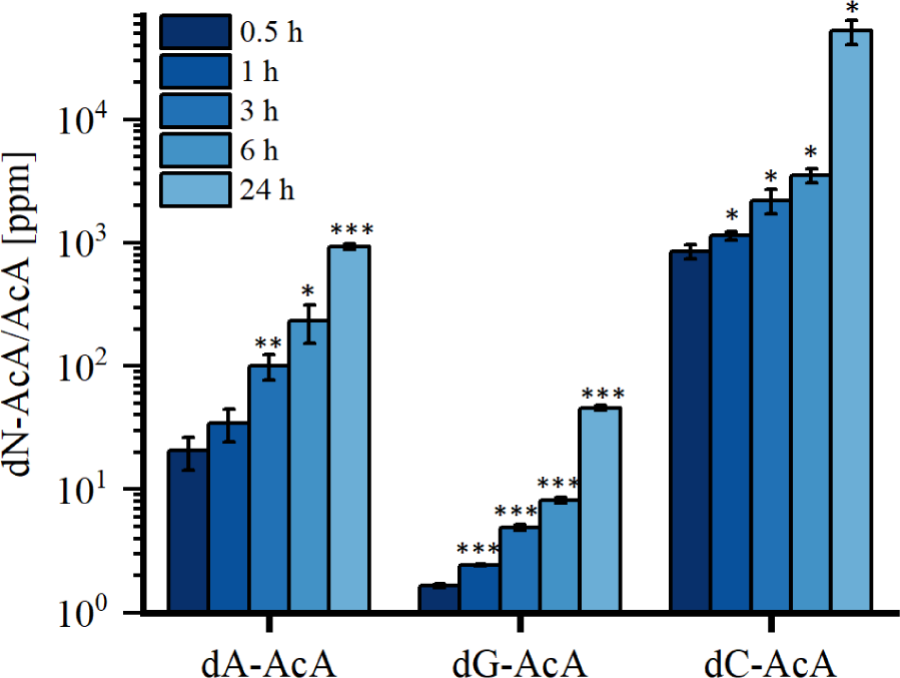
Ratio of dN-AcA/AcA [ppm] of AcA to dA-, dG- or dC-AcA after incubation with isolated DNA from pRH. *n* = 3, mean ± standard deviation. Significances were tested against the next lower unit using unpaired, one-tailed *t* test with (*) *p* < 0.05 significant, (**) *p* < 0.01 highly significant, (***) *p* < 0.001 highly significant. AcA: 3-acetylacrolein, dA: 2′-deoxyadenosine, dC: 2′-deoxycytosine, dG: 2′-deoxyguanosine, dN: nucleoside.

Based on the time- and dose-dependent formation of the three identified adducts dG-AcA, dA-AcA, and dC-AcA (Figure 4), the method can be assessed as suitable.

The mass spectrometric detection of two DNA adducts (dA-AcA, dG-AcA) is sensitive and quantitative while the determination of dC-AcA can be categorized as semiquantitative. With regard to reactivity, a steady, significant increase in adduct levels can already be observed after a short time of 0.5 h (30 min). The time-dependent conversion rate reflects continuous formation.

### Reactivity of AcA and 2-MF with pRH

Afterward the occurrence of the DNA adducts dA-AcA, dG-AcA, and dC-AcA was investigated at the cellular level. pRH were incubated for 1, 6, 18, 24, and 48 h with 2-MF (10, 50, 100, 500, 1000 μM) or AcA (0.1, 0.5, 1, 5 μM). The DNA was extracted from the cells and enzymatically hydrolyzed. The DNA adducts were analyzed by UHPLC-MS/MS using the isotope-labeled internal standards. None of the three DNA adducts were detected at any of the concentrations and time points used. In the case of dC-AcA, which can be determined semiquantitatively, no difference to the background in the solvent or negative control were detected.

The recovery of spiked samples (processed DNA from pRH incubated with 2-MF or AcA) was 80–95%. From 1 nM dC-AcA used, the signal of the molecular ion could be distinguished from the background of the negative control.

## Discussion

The oxidation of 2-MF using DMDO required the prior synthesis of the oxidizing agent with a sometimes highly reactive intermediate product in a lower yield. However, AcA was isolated as a pure substance, which was the prerequisite for subsequent stability, reaction, and incubation studies. DMDO has been increasingly used in the literature to chemically simulate the enzymatic (CYP-mediated) monooxygenation of a substance in xenobiotic metabolism. For example, DMDO was used for the oxidation of furan to generate BDA in acetone-containing solution.^26,30,31^ Another decisive advantage is that excessive DMDO reacts to acetone, which can be easily removed. At the beginning of this work, 2-MF was oxidized with *m*-chloroperbenzoic acid in accordance with the synthesis instructions of Ravindranath et al. (1984),^13^ but no complete separation of the byproducts could be achieved. Although with a different oxidizing agent, they already showed that AcA can in principle be generated from DHDMMF or direct oxidation of 2-MF by DMDO. The ^1^H-NMR data were in accordance with the data already published in 1984 by Ravindranath.^13^

Stability test of AcA in D_2_O revealed a hydration as already described for other α-,ß-unsaturated aldehydes from alkylfurans. In the literature such a hydration is also known for *cis*/*trans*-BDA^32,33^ (ratio 1:1 or 2:1) as well as 4-oxo-2-nonenal (4ONE).^34^

As shown before in Schäfer et al. 2024^14^ the reactivity of AcA toward thiol and amino groups in protein was investigated via the reaction with *N*-α-acetylated cysteine (AcCys) and lysine (AcLys). In contrast to the DNA adducts, the instantaneous reaction of AcA with the AcLys or AcCys resulted in steady adduct levels already after 1 min.

*In chemico*, the adducts of the three nucleosides and AcA were successfully synthesized. The structure of dC-AcA adduct was already reported by Rentel et al. (2005).^35^ The ^1^H- and ^13^C-NMR and MS data agreed with this reference whereas for the dG-AcA adduct, the ^1^H- and ^13^C-NMR and MS data were in agreement with Hecht et al. (1992).^36^ The adduct of AcA with dA has not yet been described in the literature, so only the comparison of dA-AcA with dG-AcA and dC-AcA was done here to compare the synthesis product. After successful synthesis of the three DNA adducts, a sensitive UHPLC-ESI^+^-MS/MS method was developed for simultaneous quantification of the three nucleoside adducts. For the establishment of a SIDA, ^15^*N*_5_-dA-AcA and^15^*N*_5_-dG-AcA were used as internal standards.

AcA was reactive toward the nucleosides dA, dG and dC, whereby a comparable formation mechanism was concluded based on the uniform adduct patterns. The postulated initial step of the reaction mechanism would also explain why AcA does not form adducts with 2′-deoxythymidine, which has no exocyclic amino group. The following hypothesis was put forward: adduct formation is initiated by the nucleophilic attack of the exocyclic amino group of the base to the aldehyde function of AcA in combination with the Michael addition of an endocyclic amide moiety to the sp^2^-hybridized C2 atom of AcA.^35−37^ In addition to the dehydrated end product, the cyclized half ketal was detected as a precursor by mass spectrometry and as a UV/Vis-active compound.

The next step was to investigate the reactivity of AcA with isolated DNA. On the one hand, methodological questions were to be clarified, such as the hydrolysis of the DNA into its monomers to release the nucleoside adducts. On the other hand, the aim was to investigate the relative distribution of the three adducts among each other. Conclusions on the formation rate could be drawn from their time- and dose-dependent formation. In most of the literature on the synthesis of DNA adducts, studies on adduct formation after lipid peroxidation were carried out. Here it was observed that 4-oxo-2-alkenals resulting from the oxidative degradation of fatty acids react with free nucleosides and isolated DNA. The resulting adducts pattern corresponded to the dA-, dG-, or dC-AcA characterized here. The chain length substituted on the 5-ring varied depending on the fatty acid used or the localization of its unsaturated bond.^37−39^

In untreated salmon DNA the negative control already contained DNA adducts of AcA. As the salmon DNA was acquired commercially, we can only speculate about the cause of the DNA adducts in the untreated control. A high level of contamination as found here speaks less in favor of contamination of the animal source than for a process- or storage-related origin. In addition, the salmon DNA originated from spermatozoids, which first represent a well-protected cell type and second should in principle have a reduced exposure to 2-MF (as a heat-induced food contaminant). In addition, DNA from untreated pRH did not show any such adducts. So, DNA from pRH was used and incubated with AcA. The ratio of dN-AcA/AcA in Figure 4 answers the question of how much of the AcA used was converted to one of the DNA adducts. Here, the adduct level was given in relation to the amount of AcA used (100 μM) in [ppm]. After 24 h, AcA was converted to 376 ± 29 ppm in dA-AcA and 24 ± 2 ppm in dG-AcA. The conversion to 27 010 ± 6670 ppm dC-AcA is to be regarded as a semiquantitative value.

An important parameter for assessing the genotoxic potential is the binding site of the adduct on the nucleoside. In particular, the base regions that form the hydrogen bonds to the complementary DNA base must be emphasized. As a result of a base mismatch that is not recognized by DNA repair, it can establish itself as a mutation after replication. Such critical regions of the DNA bases are the *N*^1^ and *N*^6^ position of dA, *N*^1^, *N*^2^ and *O*^6^ position of dG as well as the *N*^3^, *N*^4^ and *O*^2^ position of dC.^40,41^ Such regions are affected in all three DNA adducts with AcA, which suggests a mutagenic potential. This emphasizes the need to investigate the formation of AcA-DNA adducts in vitro and, if necessary, further in vivo.

Consequently, pRH were incubated with 2-MF or AcA. None of the three DNA adducts were detected at any of the concentrations and time points used. Since the content of extracted DNA was determined for each sample using UV/Vis spectroscopy to adjust the quantity, incorrect extraction can be ruled out. The experiments on the reactivity of AcA with isolated DNA from untreated pRH confirm that potentially formed adducts can be successfully released by means of enzymatic hydrolysis. It cannot be ruled out that disintegration of the adducts occurred during isolation of the DNA from the cell. This option was considered unlikely, as the adducts characterized in this work, as well as comparable cyclic etheno adducts, are described in the literature as stable.^36,42^ With the exclusion of methological and processing-related errors, the conclusion is that no dA-AcA, dG-AcA or dC-AcA were formed in pRH up to the limits of determination or recovery.

CYPs that metabolize 2-MF are primarily integrated into the membrane of the endoplasmic reticulum. Therefore, AcA is released as a metabolite of 2-MF in the cytosol, where nucleophiles are present as potential binding partners. If the potential to bind to these is high, the probability of diffusion into the nucleus to the DNA might be reduced. If reactive molecules such as other 2-oxo-4-alkenyls are stabilized via a longer hydrocarbon chain, they are more likely to reach and bind to DNA. The fact that AcLys-AcA was detected here points to a partial detoxification of AcA.^14^ Although the depletion of amino acids or peptides is also highly damaging to cells, the more problematic DNA adduct formation is initially prevented or reduced.

Comparable behavior has been described for furan with regard to a high reactivity toward isolated DNA and no detection of corresponding adducts in vivo. Analogous to AcA, the formation of etheno adducts on nucleosides and isolated DNA was observed for BDA.^43,44^ When rats were administered 9.2 mg/kg bw furan in vivo either in a single dose or spread over 360 days, the BDA adducts could not be detected in hepatic DNA.^45^ Our findings are in line with Peterson, discussing that furan metabolites showed a higher reactivity toward proteins than DNA.^46^ The author state that there might be competing metabolic pathways.

In conclusion, for the risk assessment of 2-MF, it can be stated that the DNA adducts formed *in chemico* (dA-AcA, dG-AcA, dC-AcA) were not formed in pRH up to the limit of quantification (0.05 nM). However, it cannot be ruled out that deviating adducts could be formed by exposure to 2-MF. It is therefore necessary to test whether other secondary metabolites such as FFA are formed by hydroxylation of the methyl group via phase I of xenobiotic metabolism. The formation of DNA adducts in vivo is known for FFA.^47,48^ Consequently, the comparison of mechanistic properties between 2-MF and furan would be invalid due to the difference in the crucial structural element.

## Supporting Information Available

Table S1: ^1^H-NMR signals of *cis*-AcA and *cis*-AcA–OH_2_ and their relative presence in different solvents. Figure S1:Characterization of dA-AcA. ^1^H-NMR spectrum (400 MHz) of dA-AcA in DMSO-*d*_6_. Table S2: Characterization of dA-AcA. Identification of dA-AcA via NMR and MS. In DMSO-*d*_6_ with^1^H-NMR (400 MHz) and ^13^C-NMR (101 MHz). Figure S2: Characterization of dA-AcA. HPLC-ESI^+^-MS^2^ spectrum of dA-AcA with postulated, characteristic fragment structures. Table S3: Characterization of dA-AcA. Precursor ions and fragments [*m*/*z*] in product ion scan (MS^2^) of dA-AcA and ^15^*N*_5_-dA-AcA. Figure S3: Characterization of dG-AcA. ^1^H-NMR spectrum (400 MHz) of dG-AcA in DMSO-*d*_6_. Table S4: Characterization of dG-AcA. Identification of dG-AcA via NMR and MS with literature comparison. Figure S4: Characterization of dG-AcA. HPLC-ESI^+^-MS^2^ spectrum of dG-AcA with postulated, characteristic fragment structures. Table S5: Characterization of dG-AcA. Precursor ions and fragments [*m*/*z*] in product ion scan (MS^2^) of dG-AcA and ^15^*N*_5_-dG-AcA. Figure S5: Characterization of dC-AcA. ^1^H-NMR sprectrum (400 MHz) of dC-AcA in DMSO-*d*_6_. Table S6: Characterization of dC-AcA. ^1^H- and ^13^C-NMR signals of dC-AcA from the synthesis compared to literature data. Figure S6: Characterization of dC-AcA. Identification of dC-AcA. I.) Signal assignment of ^1^H- and ^13^C-NMR spectra II.) Fragmentation pattern from LC-ESI^+^-MS/MS measurements. Figure S7: Characterization of dC-AcA. HPLC-ESI^+^-MS^2^ spectrum of dC-AcA with postulated, characterized fragmentation pattern. Table S7: Approach for in chemico reactions of desoxynucleosides with AcA from synthesis with DMDO. Table S8: Approach for investigating the reactivity of AcA with isolated DNA. Table S9: MS specific parameters for quantification of DNA adducts dA-AcA, dG-AcA and dC-AcA with [^15^*N*_5_] dA-AcA and [^15^*N*_5_]-dG-AcA. Figure S8: Contents of dA-AcA (A), dG-AcA (B) and dC-AcA (C) in untreated salmon DNA without AcA treatment (PDF)

## Abbreviations

AcA: 3-acetylacrolein, 4-oxopent-2-enal
2-MF: 2-methylfuran
pRH: primary rat hepatocytes
dG: 2′-deoxyguanosine
dC: 2′-deoxycytosine
dT: 2′-deoxythymidine
dA: 2′-deoxyadenosine
dR: desoxyribose
