# Supplementary material for: Reactivity of the 2-Methylfuran Phase I Metabolite 3-Acetylacrolein Toward DNA
Source: J Agric Food Chem. 2024 Nov 4;72(45):25319–29. doi: 10.1021/acs.jafc.4c07280 (PMC11565790; doi:10.1021/acs.jafc.4c07280)
Supplement: Supplementary file 1 — jf4c07280_si_001.pdf [file jf4c07280_si_001.pdf]

Supporting Information

Reactivity of the 2-methylfuran phase I metabolite acetylacrolein towards DNA

Verena Schäfer, Simone Stegmüller, Hanna Becker, Elke Richling*

Department of Chemistry, Division of Food Chemistry and Toxicology, University of Kaiserslautern-Landau, Kaiserslautern, Germany

Corresponding author's contact information: Prof. Elke Richling,

e-mail: elke.richling@chem.rptu.de

Table S1: ^{1}H-NMR signals of *cis*-AcA and *cis*-AcA-OH_2 and their relative presence in different solvents.

Figure S1: Characterisation of dA-AcA. ^{1}H-NMR spectrum (400 MHz) of dA-AcA in DMSO-d_6.

Table S2: Characterisation of dA-AcA. Identification of dA-AcA via NMR and MS. In DMSO-d_6 with ^{1}H-NMR (400 MHz) and ^{13}C-NMR (101 MHz).

Figure S2: Characterisation of dA-AcA. HPLC-ESI$^+$-MS^2 spectrum of dA-AcA with postulated, characteristic fragment structures.

Table S3: Characterisation of dA-AcA. Precursor ions and fragments [*m*/*z*] in product ion scan (MS^2) of dA-AcA and $^{15}N_5$-dA-AcA.

Figure S3: Characterisation of dG-AcA. ^{1}H-NMR spectrum (400 MHz) of dG-AcA in DMSO-d_6.

Table S4: Characterisation of dG-AcA. Identification of dG-AcA via NMR and MS with literature comparison.

Figure S4: Characterisation of dG-AcA. HPLC-ESI$^+$-MS^2 spectrum of dG-AcA with postulated, characteristic fragment structures.

Table S5: Characterisation of dG-AcA. Precursor ions and fragments [*m*/*z*] in product ion scan (MS^2) of dG-AcA and $^{15}N_5$-dG-AcA.

Figure S5: Characterisation of dC-AcA. ^{1}H-NMR sprectrum (400 MHz) of dC-AcA in DMSO-d_6.

Table S6: Characterisation of dC-AcA. ^{1}H- and ^{13}C-NMR signals of dC-AcA from the synthesis compared to literature data.

Figure S6: Characterisation of dC-AcA. Identification of dC-AcA. I.) Signal assignment of ^{1}H- and ^{13}C-NMR spectra II.) Fragmentation pattern from LC-ESI^{+}-MS/MS measurements.

Figure S7: Characterisation of dC-AcA. HPLC-ESI^{+}-MS^{2} spectrum of dC-AcA with postulated, characterized fragmentation pattern.

Table S7: Approach for in chemico reactions of desoxynucleosides with AcA from synthesis with DMDO.

Table S8: Approach for investigating the reactivity of AcA with isolated DNA.

Table S9: MS specific parameters for quantification of DNA adducts dA-AcA, dG-AcA and dC-AcA with [$^{15}N_5$] dA-AcA and [$^{15}N_5$]-dG-AcA.

Figure S8: Contents of dA-AcA (A), dG-AcA (B) and dC-AcA (C) in untreated salmon DNA without AcA treatment.

Table S1: ^{1}H-NMR signals of *cis*-AcA and *cis*-AcA-OH_2 and their relative presence in different solvents. Mix: Presence as diastereomers. Ratios of diastereomer mixtures were determined by integrating the protons in the ^{1}H-NMR. AcA: 3-acetylacrolein.

	cis-AcA-OH_2<br>600 MHz, D_2O				*cis*-AcA<br>400 MHz, DMSO-d_6			
Signal	δ [ppm]	∫	m	J [Hz]	δ [ppm]	∫	m	J [Hz]
A	6.16	2H	ddd	5.8<br>2.8<br>1.2	9.94	1H	d	6.9 ($3J^{A,C}$)
B	6.07	1H	s		7.20	1H	d	11.8 ($3J^{B,C}$)
C	6.01	2H	d	5.8	6.22	1H	dd	11.8 ($3J^{B,C}$)<br>6.9 ($3J^{A,C}$)
D	5.86	1H	s		2.34	3H	s	
E	1.57	3H	s					
F	1.49	3H	s					
	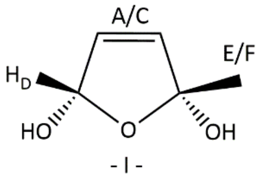		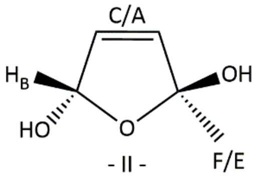		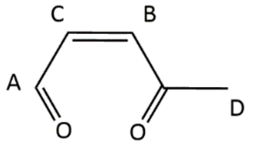			
Mix:	*cis*-AcA:*cis*-AcAI-OH_2: *cis*-AcAII-OH_2 (0.2:1:1)							

Characterisation of dA-AcA

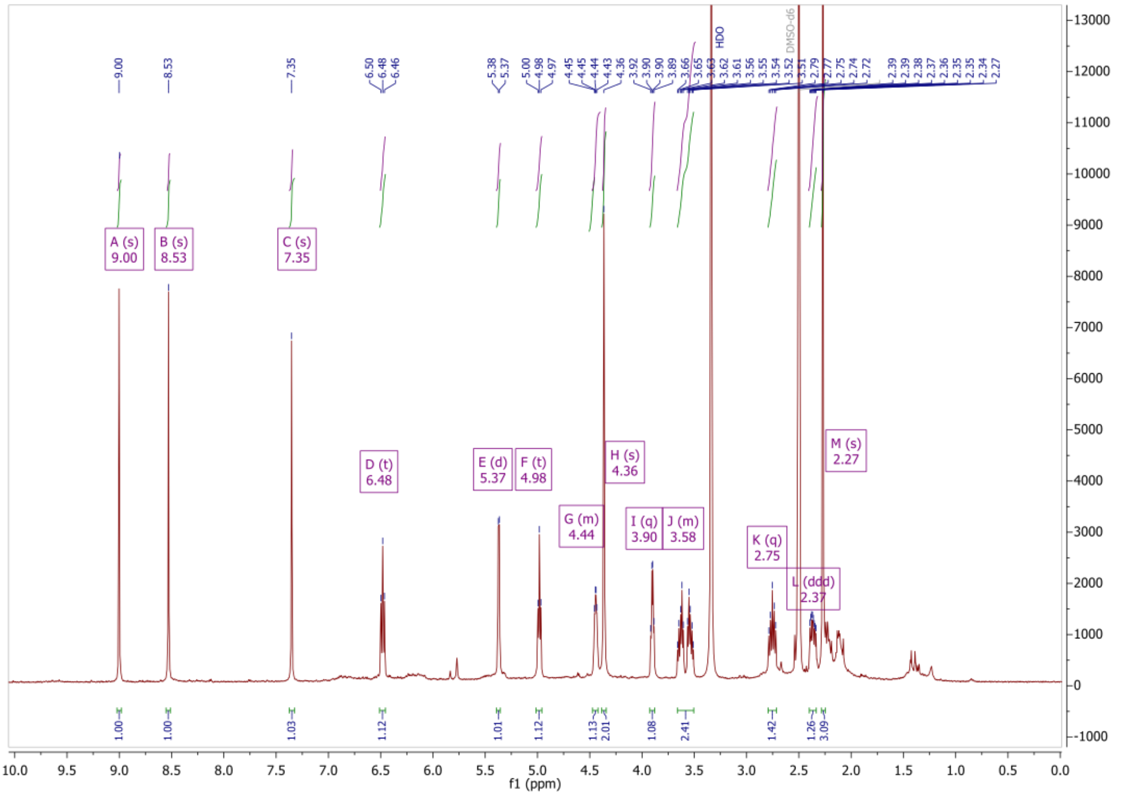

Figure S1: ^{1}H-NMR spectrum (400 MHz) of dA-AcA in DMSO-d_6. The assignment of the signals is shown in Table S2. AcA: 3-acetylacrolein, dA: 2′-desoxyadenosin.

Table S2: Identification of dA-AcA via NMR and MS. In DMSO-d_6 with ^{1}H-NMR (400 MHz) and ^{13}C-NMR (101 MHz). Characteristic main fragments by using ESI^{+}-MS/MS. *) Theoretical estimation because of overlapping with solvent signal. AcA: 3-acetylacrolein, dA: 2′-desoxyadenosin, *m*/*z*: mass to charge ratio.

^{1}H-NMR					^{13}C-NMR		Signal assignment of
Signal	∫	Δ [ppm]	*m*	*J* [Hz]	Signal	Δ [ppm]	NMR signals and MS fragmentation
A	1H	9.00	s		a	204.45	
B	1H	8.53	s		b	140.77	
C	1H	7.35	s		c	139.91	
D	1H	6.48	t	6.8	d	137.90	
E	1H	5.37	d	4.1	e	135.88	
F	1H	4.98	t	5.5	f	131.98	
G	1H	4.45	m		g	123.11	
H	2H	4.36	s		h	118.62	
I	1H	3.90	q	4.6	i	88.03	
J	2H	3.58	m		j	84.06	
K	1H	2.75	q	13.2	k	70.78	
				6.1	l	61.73	
L	1H	2.37	ddd	13.2	l	61.73	
				6.2	m	~40*	
				3.3	n	37.97	
M	3H	2.27	s		o	29.46	

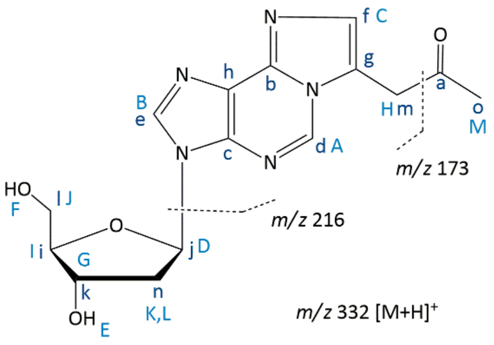

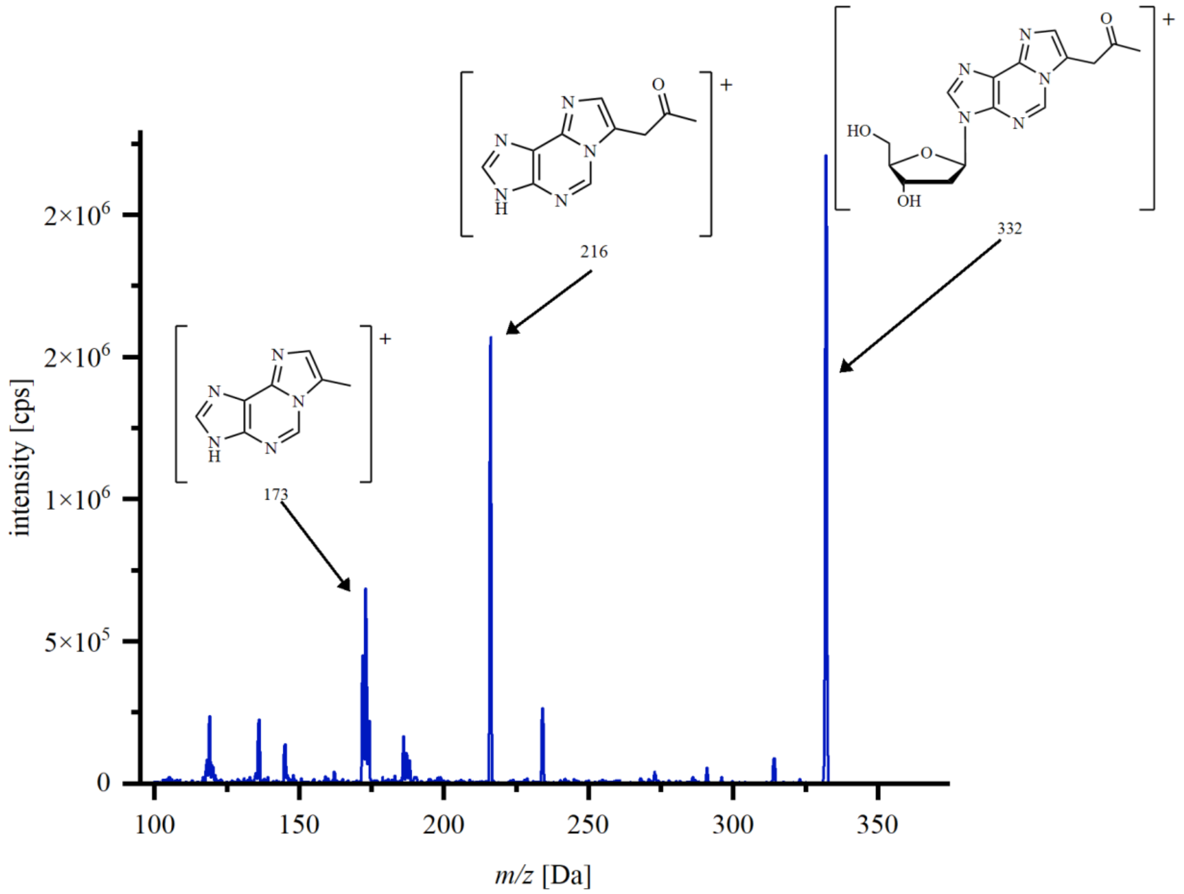

Figure S2: HPLC-ESI$^+$-MS^2 spectrum of dA-AcA with postulated, characteristic fragment structures. AcA: 3-acetylacrolein, dA: 2′-desoxyadenosin, *m/z:* mass to charge ratio.

Table S3: Precursor ions and fragments [*m/z*] in product ion scan (MS^2) of dA-AcA and $^{15}N_5$-dA-AcA. Ac: Acetyl, AcA: 3-acetylacrolein, dA: 2′-desoxyadenosin, dR: 2′-desoxyribose, *m/z:* mass to charge ratio, PropCO: Isopropenyloxyl.

Substanz	$[M+H]^+$	$[M-H_2O]^+$	$[M-dR]^+$	$[M-dR-CO_2]^+$	$[M-dR-Ac]^+$	$[dA+H]^+$
dA-AcA	332.0	314.0	216.2	186.0	173.0	136.2
$^{15}N_5$-dA-AcA	337.2	319.2	221.0	191.0	179.2	141.0

Characterisation of dG-AcA

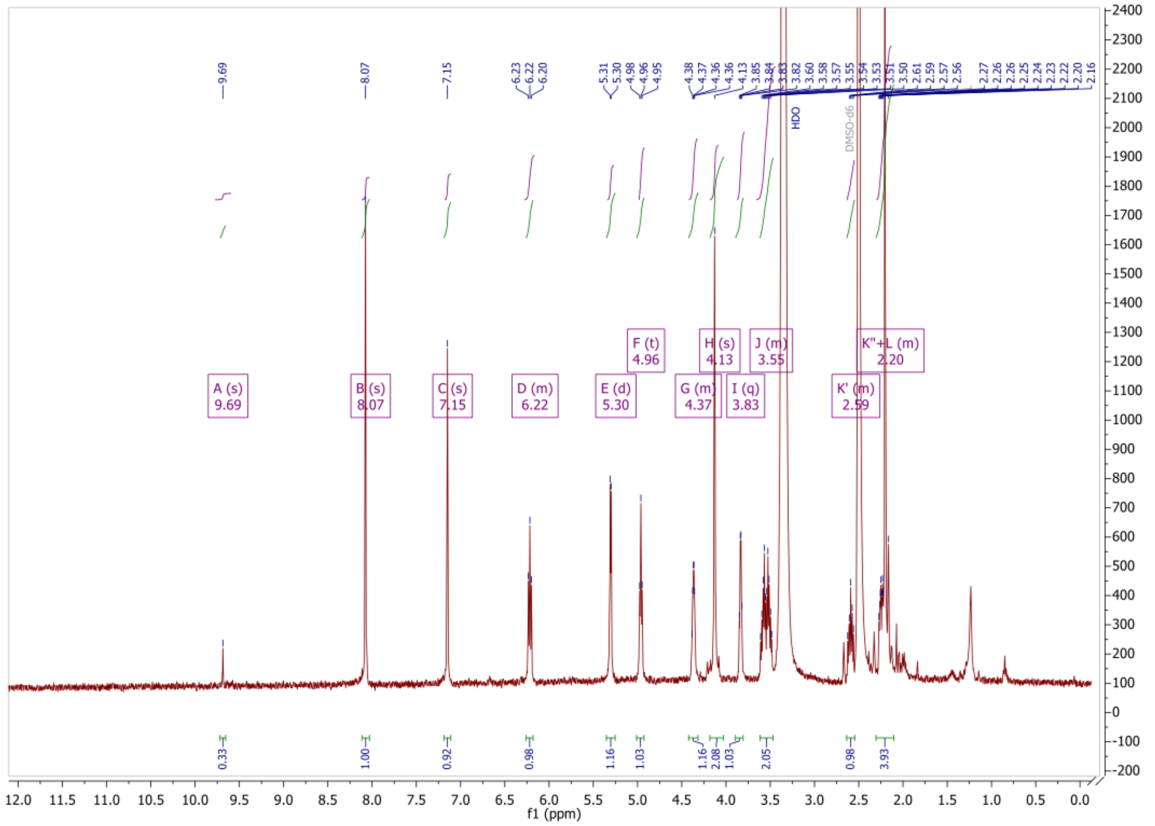

Figure S3: ^{1}H-NMR spectrum (400 MHz) of dG-AcA in DMSO-d_6. The assignment of the signals is shown in Table S4. AcA: 3-acetylacrolein, dG: 2′-desoxyguanosin.

Table S4: Identification of dG-AcA via NMR and MS with literature comparison. Syn: own synthesis in DMSO-d_6 with ^{1}H-NMR (400 MHz) and characteristic main fragments by using ESI^{+}-MS/MS, Ref.: Reference literature in DMSO-d_6 was partially only available as a spectrum, which is why the δ marked with (~) was only estimated (Hecht et al., 1992). *) amide-bound proton with low signal intensity. AcA: 3-acetylacrolein, dG: 2′-desoxyguanosin, *m/z*: mass to charge ratio.

Signal	Syn. ∫	Syn. δ [ppm]	Syn. *m*	Syn. *J* [Hz]	Ref. ~ δ [ppm]	Signal assignment NMR signals and MS fragmentation
A	*	9.69	s			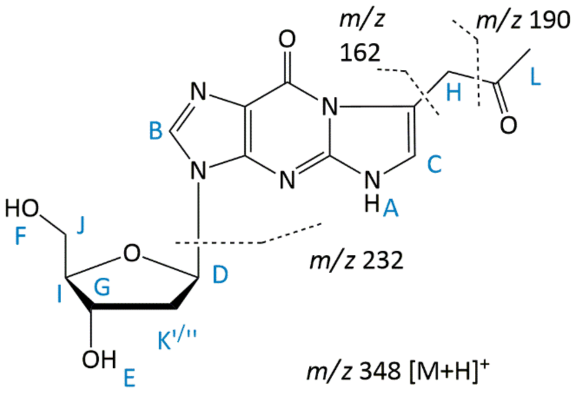
B	1H	8.07	s		8.06	
C	1H	7.15	s		7.13	
D	1H	6.22	m		~6·2	
E	1H	5.30	d	4,0		
F	1H	4.96	t	5,5		
G	1H	4.37	m		~4.4	
H	2H	4.13	s		4.10	
I	1H	3.83	q	4,5	~3.9	
J	2H	3.55	m		~3.5	
K’	1H	2.59	m		~2.6	
K’’+L	4H	2.20	m+s		2.20	

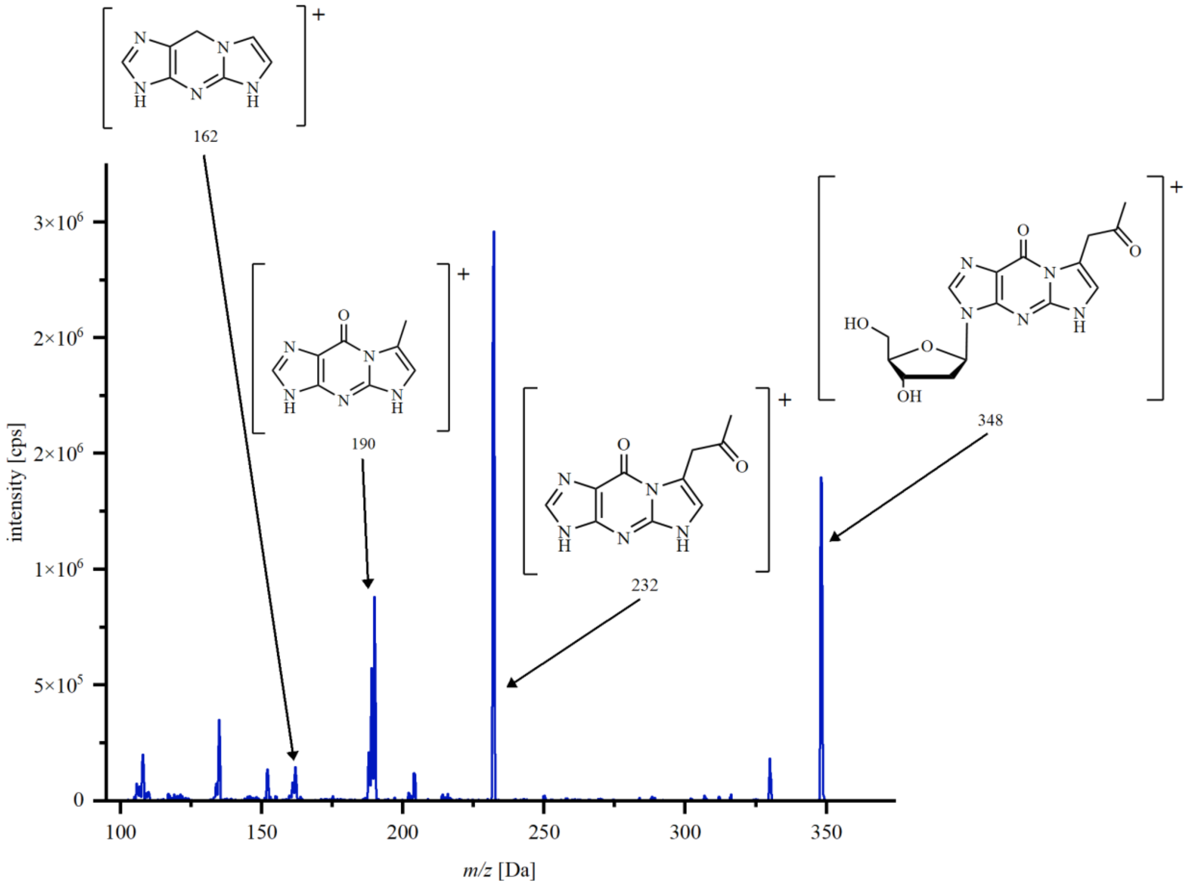

Figure S4: HPLC-ESI$^+$-MS^2 spectrum of dG-AcA with postulated, characteristic fragment structures. AcA: 3-acetylacrolein, dG: 2′-desoxyguanosin, *m/z:* mass to charge ratio.

Table S5: Precursor ions and fragments [*m/z*] in product ion scan (MS^2) of dG-AcA and $^{15}N_5$-dG-AcA. Ac: Acetyl, AcA: 3-acetylacrolein, dG: 2′-desoxyguanosin, dR: 2′-desoxyribose, *m/z*: mass to charge ratio, PropCO: Isopropenyloxyl.

Substanz	$[M+H]^+$	$[M-H_2O]^+$	$[M-dR]^+$	$[M-dR-Ac]^+$	$[M-dR-PropCO]^+$	Fragments of ring system	
dG-AcA	348.2	330.2	232.2	190.0	162.0	152.0	135.0
$^{15}N_5$-dG-AcA	353.2	335.0	237.0	195.0	167.0		139.0

Characterisation of dC-AcA

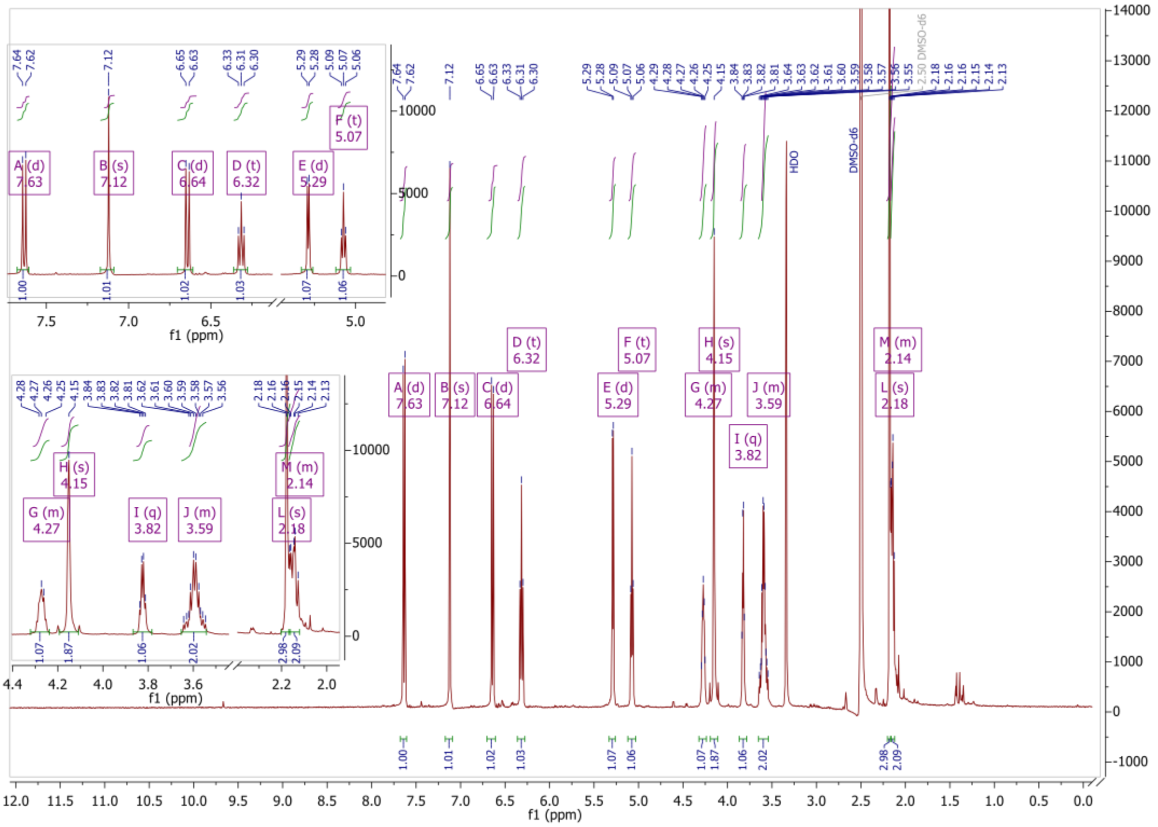

Figure S5: ^{1}H-NMR sprectrum (400 MHz) of dC-AcA in DMSO-d_6. The assignment of the signals is shown in Table S6 and Figure S6. AcA: 3-acetylacrolein, dC: 2′-desoxycytosin.

Table S6: ^{1}H- and ^{13}C-NMR signals of dC-AcA from the synthesis compared to literature data. Own synthesis in DMSO-d_6 with ^{1}H-NMR (400 MHz) and ^{13}C-NMR (101 MHz), Reference literature in DMSO-d_6 (Rentel et al., 2005). AcA: 3-acetylacrolein, dC: 2′-desoxycytosin.

	**^{1}H-NMR**								**^{13}C-NMR**		
	Own synthesis				Reference literature					Syn.	Ref.
Signal	∫	*δ* [ppm]	*m*	*J* [Hz]	∫	*δ* [ppm]	*m*	*J* [Hz]	Signal	*δ* [ppm]	*δ* [ppm]
A	1H	7.63	d	8.0	1H	7.63	d	8.0	a	204.46	204.2
B	1H	7.12	s		1H	7.12	s		b	146.88	146.8
C	1H	6.64	d	8.0	1H	6.64	d	7.9	c	145.22	145.1
D	1H	6.32	t	6.8	1H	6.31	t	6.7	d	132.68	132.6
E	1H	5.29	d	4.2	1H	5.28	d	4.2	e	127.92	127.8
F	1H	5.07	t	5.1	1H	5.06	t	5.2	f	123.09	123.0
G	1H	4.27	m		1H	4.27	m		g	98.94	98.8
H	2H	4.16	s		2H	4.15	s		h	87.71	87.6
I	1H	3.84	q	3.5	1H	3.83	m		i	84.80	84.7
J	2H	3.60	m		2H	3.60	m		j	70.45	70.3
K	3H	2.19	s		5H	2.14	m		k	61.31	61.2
L	2H	2.14	m						l	40.14	40.0
									m	39.83	39.7
									n	29.37	29.2

For comparison, the reactant dC showed the following ^{1}H-NMR signals: ^{1}H-NMR (400 MHz, DMSO-d_6) *δ* 7.78 (d, *J* = 7.4Hz, 1H), 7.10 (d, *J* = 27.1Hz, 2H), 6.15 (t, J = 6.6 1H), 5.71 (d, *J* = 7.4Hz, 1H), 5.20 (d, *J* = 4.2Hz, 1H), 4.97 (t, *J* = 5.3Hz, 1H), 4.22–4.13 (m, 1H), 3.75 (q, *J* = 3.7 Hz, 1H), 3.62 – 3.46 (m, *J* = 5.0 Hz, 2H), 2.14–2.04 (m, 1H), 1.92 (p, *J* = 13.3, 6.2Hz, 1H).

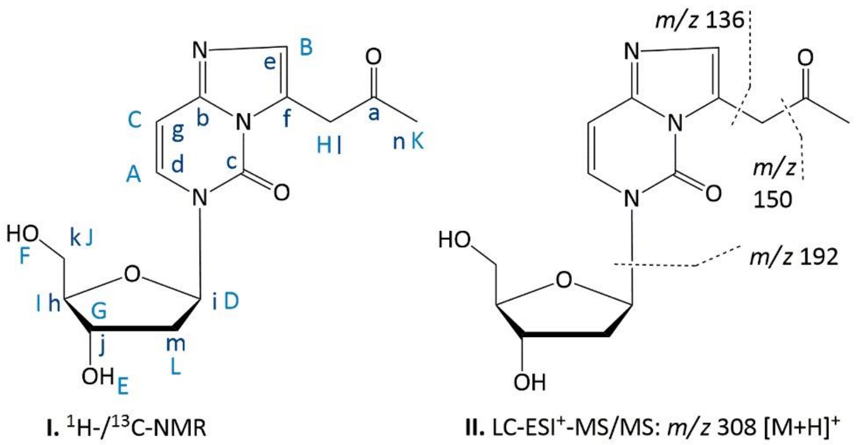

Figure S6: Identification of dC-AcA. I.) Signal assignment of ^{1}H- and ^{13}C-NMR spectra according to the signals in Table S6 II.) Fragmentation pattern from LC-ESI$^+$-MS/MS measurements. AcA: 3-acetylacrolein, dC: 2′-desoxycytosin, *m*/*z*: mass to charge ratio.

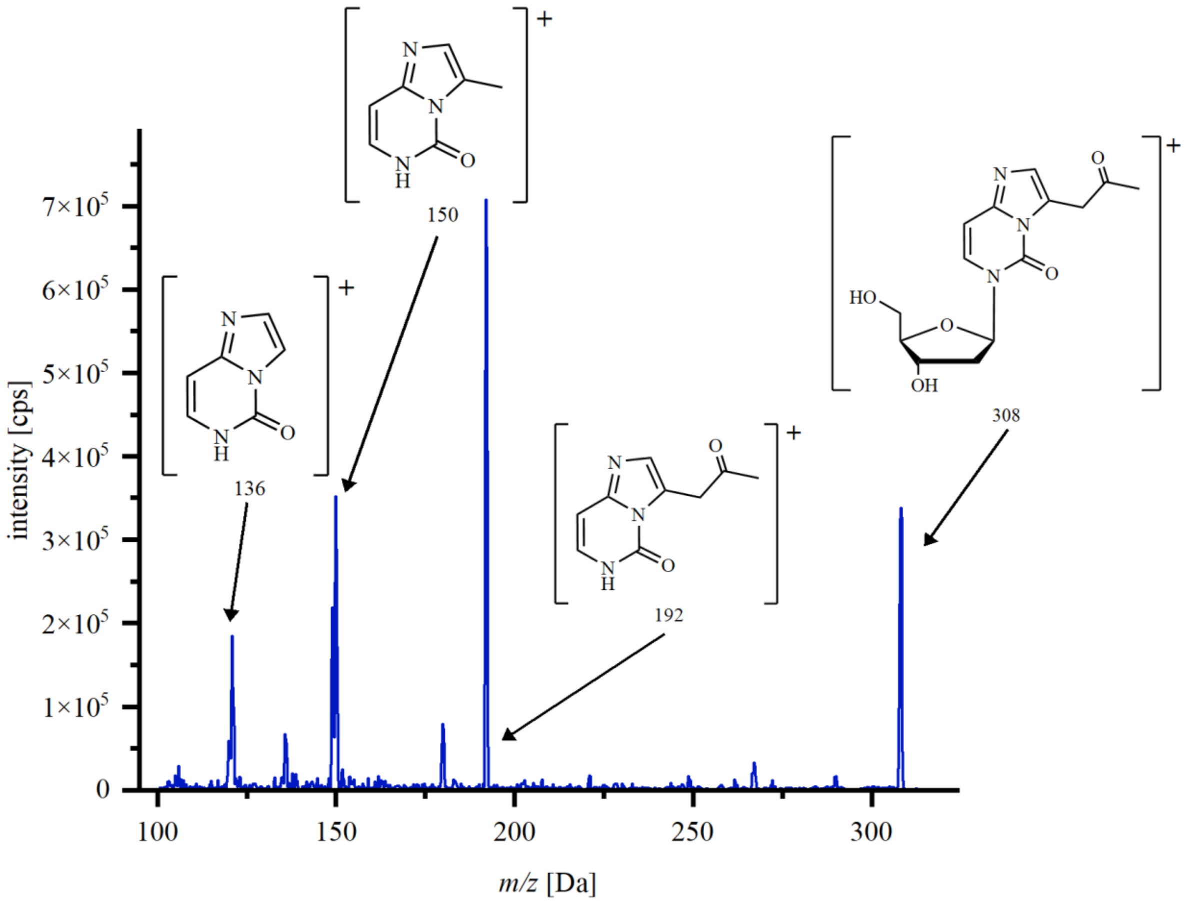

Figure S7: HPLC-ESI$^+$-MS^2 spectrum of dC-AcA with postulated, characterized fragmentation pattern. AcA: 3-acetylacrolein, dC: 2′-desoxycytidin, *m/z:* mass to charge ratio.

Table S7: Approach for reaction in chemico with AcA from synthesis with DMDO. *) Buffer: K_2HPO_4 (SS 500 mM), Tris-HCl (SS 500 mM) or $(NH_4)_2CO_3$ (SS 100 mM) with pH 5, 7.4 or 9. **) Test substance: 3-acetylacrolein (AcA) or 2-methylfuran (MF). ***) Reactant: 2′-deoxyadenosine (dA), 2′-deoxycytosine (dC), 2′-deoxyguanosine (dG) or 2′-deoxythymidine (dT). DMDO: dimethyldioxirane, SS: stock solution, DF: dilution factor.

substances	*c*(SS)	*c*	*n*	DF from SS	V(SS)/500 µL reaction mixture
	[mM]	[mM]	[µmol]		[µL]
H_2O					150
buffer*	100 | 500	10 | 50		0.1	50
test substance**	100	10	5	0.1	50
reactant***	10	5	2.5	0.5	250

Table S8: Approach for investigating the reactivity of AcA with isolated DNA. *) Buffer: K_2HPO_4. AcA: 3-acetylacrolein, RS: reaction solution, DF: dilution factor, ID: intermediate dilution.

substance	*c*(RS)	*c*(ID)	DF from ID	V(ID) [µL]/100 µL RS
DNA	370 µg/mL	925 µg/mL	0.4	54
AcA	0.1–1000 µM	10·*c*(RS)	0.1	10
*buffer**	0.05 M	0.5 M	0.1	10
H_2O, *without buffer*				36
with buffer				26

Table S9: MS specific parameters for quantification of DNA adducts dA-AcA, dG-AcA and dC-AcA with [$^{15}N_5$]-dA-AcA and [$^{15}N_5$]-dG-AcA. Underlined mass transitions were used as quantifiers. CAD: collisionally activated dissociation, CE: collision energy, CEP: collision cell entrance potential, CUR: curtain gas, CXP: cell exit potential, DP: declustering potential, dtime: dwell time, EP: entrance potential, GS1: nebulizer gas, GS2: heater gas, IS: ion spray voltage, Q1: quadrupol 1, Q3: quadrupol 3, TEMP: ion source temperature. AcA: 3-acetylacrolein, dA: 2′-desoxyadenosin, dC: 2′-desoxycytosin, dG: 2′-desoxyguanosin, *m*/*z*: mass-to-charge ratios.

Substance specific parameters							
	Q1 [*m*/*z*]	Q3 [*m*/*z*]	dtime [msec]	DP [V]	EP [V]	CE [eV]	CXP [V]
dA-AcA	<u>332.026</u>	<u>216.0</u>	70	31	10	31	14
	332.026	173.0	70	31	10	53	14
dG-AcA	<u>348.031</u>	<u>232.1</u>	70	51	10	31	26
	348.031	190.0	70	51	10	45	12
dC-AcA	<u>308.043</u>	<u>192.0</u>	70	76	10	19	14
	308.043	150.1	70	76	10	45	14
	308.043	121.1	70	76	10	63	14
$^{15}N_5$-dG-AcA	<u>353.024</u>	<u>237.0</u>	70	131	10	31	16
	353.024	195.0	70	131	10	45	18
$^{15}N_5$-dA-AcA	<u>336.917</u>	<u>221.0</u>	70	46	10	37	16
	336.917	179.1	70	46	10	51	16

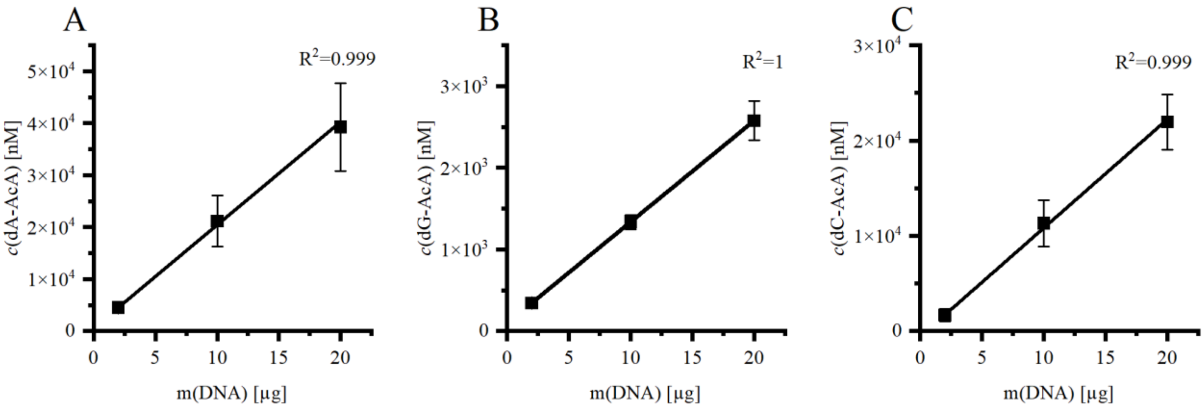

Figure S8: Contents of dA-AcA (A), dG-AcA (B) and dC-AcA (C) in untreated salmon DNA without AcA treatment. Determined via HPLC-ESI$^+$-MS/MS, MRM method. *n*=3, mean standard deviation. AcA: 3-acetylacrolein, dA: 2′-deoxyadenosine, dC: 2′-deoxycytosine, dG: 2′-deoxyguanosine.
